# Predicting muscle forces of individuals with hemiparesis following stroke

**DOI:** 10.1186/1743-0003-5-7

**Published:** 2008-02-27

**Authors:** Trisha M Kesar, Jun Ding, Anthony S Wexler, Ramu Perumal, Ryan Maladen, Stuart A Binder-Macleod

**Affiliations:** 1 301 McKinly Laboratory, Department of Physical Therapy, University of Delaware, Newark, DE 19716, USA; 2 Interdisciplinary Graduate Program in Biomechanics & Movement Science, University of Delaware, Newark, DE 19716, USA; 3 Departments of Mechanical and Aeronautical Engineering, Civil and Environmental Engineering, and Land, Air and Water Resources, University of California, Davis, CA 95616, USA

## Abstract

**Background:**

Functional electrical stimulation (FES) has been used to improve function in individuals with hemiparesis following stroke. An ideal functional electrical stimulation (FES) system needs an accurate mathematical model capable of designing subject and task-specific stimulation patterns. Such a model was previously developed in our laboratory and shown to predict the isometric forces produced by the quadriceps femoris muscles of able-bodied individuals and individuals with spinal cord injury in response to a wide range of clinically relevant stimulation frequencies and patterns. The aim of this study was to test our isometric muscle force model on the quadriceps femoris, ankle dorsiflexor, and ankle plantar-flexor muscles of individuals with post-stroke hemiparesis.

**Methods:**

Subjects were seated on a force dynamometer and isometric forces were measured in response to a range of stimulation frequencies (10 to 80-Hz) and 3 different patterns. Subject-specific model parameter values were obtained by fitting the measured force responses from 2 stimulation trains. The model parameters thus obtained were then used to obtain predicted forces for a range of frequencies and patterns. Predicted and measured forces were compared using intra-class correlation coefficients, r^2 ^values, and model error relative to the physiological error (variability of measured forces).

**Results:**

Results showed excellent agreement between measured and predicted force-time responses (r^2 ^>0.80), peak forces (ICCs>0.84), and force-time integrals (ICCs>0.82) for the quadriceps, dorsiflexor, and plantar-fexor muscles. The *model error *was within or below the +95% confidence interval of the *physiological error *for >88% comparisons between measured and predicted forces.

**Conclusion:**

Our results show that the model has potential to be incorporated as a feed-forward controller for predicting subject-specific stimulation patterns during FES.

## Introduction

According to the American Heart Association, 7.7 million people are living with the effects of stroke and over 700,000 people will experience a stroke or recurrence of a stroke annually [1]. Weakness of lower extremity muscles is a common motor impairment in individuals with hemiparesis following stroke [2]. Since 1960, functional electrical stimulation (FES) of weak or paralyzed lower extremity muscles has been used as a neuroprosthesis for the rehabilitation of individuals with hemiparesis following stroke [3,4]. FES of the lower extremity muscles can improve gait performance and aid in recovery of function in individuals with stroke [5-10], may prevent muscle atrophy [11], and play a role in the training of spinal pathways [12]. However, FES has not gained widespread application among individuals with paralysis due to limitations such as imprecise control of muscle force and the rapid onset of fatigue [13-15].

**Figure 1:**
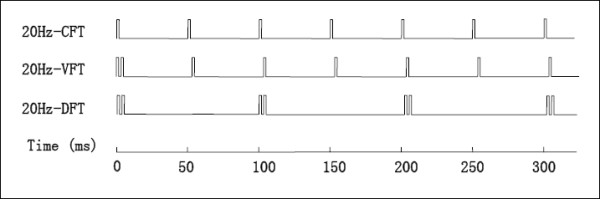
Schematic representations of the three stimulation train patterns used in this study. Top line: a 20-Hz constant-frequency train (CFT) with all the pulses spaced equally by 50-ms; Middle line: a 20-Hz variable-frequency train (VFT) with a 5-ms inter-pulse interval (doublet) inserted in the beginning of a 20-Hz CFT; Bottom line: a 20-Hz doublet-frequency train (DFT) with doublets (2 pulses with a 5-ms inter-pulse interval) spaced equally by 95-ms. All the trains were either 1-sec in duration or contained 50-pulses, whichever occurred first (See text for details).

During FES, stimulation is delivered in the form of groups of pulses called trains. At any particular intensity of stimulation, both the stimulation frequency and pattern can be varied to control muscle force. Stimulation frequency can be varied by changing the duration of the inter-pulse intervals within a stimulation train. Stimulation trains that maintain a constant inter-pulse interval throughout a train are termed constant-frequency trains (CFTs). In contrast, trains with varying inter-pulse intervals within a train are called variable-frequency trains (VFTs) [16-18]. The most common type of VFTs that have been studied consist of two closely spaced pulses with 5 to 10-ms inter-pulse interval (doublet) at the onset of a CFT [16] (Figure 1). Recently, trains consisting of regularly spaced doublets throughout the train, termed doublet-frequency trains (DFTs) have also been tested [16] (Figure 1). VFTs and DFTs have been shown to augment muscle performance compared to CFTs of comparable frequencies, especially in fatigued muscles [16,19]. However, most commercial FES stimulators only deliver CFTs.

The generation of a sufficient isometric force level for a task is a prerequisite for effective performance of an FES-elicited task. For example, to manage foot drop using FES, the electrical stimulation parameters should elicit sufficient dorsiflexor muscle force to achieve ground clearance for numerous steps. However, the frequency or pattern of the stimulation train that generates the targeted performance may vary with the task, across individuals [18], between able-bodied and paralyzed muscles [20], and with the physiological condition of the muscle, such as fatigue or muscle length [21]. Thus, numerous measurements would be needed to identify the frequency and pattern that can generate the targeted forces during FES. Mathematical models that can predict the non-linear and time-varying relationships for each subject between stimulation parameters and electrically-elicited muscle forces can help reduce the number of testing sessions. When used in conjunction with a closed-loop controller, predictive mathematical models can enable FES stimulators to deliver customized, task-specific, and subject-specific stimulation patterns while continuously adapting these patterns to the changing needs of the patient [14,22].

Our laboratory has successfully developed a Hill-based [23] phenomenological mathematical model system that predicts muscle forces in response to stimulation trains of different patterns and a range of frequencies in able-bodied subjects [24,25] and individuals with spinal cord injury [26]. A recent comparative study [27] of muscle models that can be used in FES showed that our model [28] predicted electrically-elicited forces of the soleus muscles of individuals with chronic spinal cord injury as accurately as a 2^nd ^order nonlinear model [29] and with greater accuracy than a simple linear model. Another recent study [30] comparing 7 different muscle models showed our model [28], along with the Bobet-Stein model [29] provided the best fits for ankle dorsiflexor muscle forces over a range of joint angles in able-bodied individuals. However, the model has only been tested on able-bodied subjects and individuals with spinal cord injury. In addition, for our model to be successfully incorporated in a versatile FES-controller, it must predict force responses of a variety of lower extremity muscles in different patient populations. Therefore, our purpose was to test our model on the quadriceps femoris and ankle dorsiflexor and plantar-flexor muscles of individuals with hemiparesis following stroke. The three muscles tested in our study play an important role during functional activities such as ambulation [31,32] and are commonly impaired in individuals with post-stroke hemiparesis [33-37].

### Isometric force model

Our model simplifies the various physiological processes involved in the generation of skeletal muscle force into two basic steps: muscle activation and force generation, modeled by two first-order ordinary differential equations.

$$\frac{dC_{N}}{dt}=\frac{1}{\tau_{c}}\sum_{i=1}^{n}R_{i}\exp(-\frac{t-t_{i}}{\tau_{c}})-\frac{C_{N}}{\tau_{c}},$$

whose analytical solution is given by

$$C_{N}=\sum_{i=1}^{n}R_{i}\left(\frac{t-t_{i}}{\tau_{c}}\right)\exp\left(\frac{t-t_{i}}{\tau_{c}}\right),$$

with R_i _= 1 + (R_0 _- 1)exp [-(t_i _- t_i-1_)/*τ*_c_].

Equation (1) represents the muscle activation dynamics in response to a series of electrical pulses within a stimulation train. Although a number of steps are involved between onset of stimulation and the binding of myosin filaments with actin, Ding and colleagues [25] found that it was sufficient to model the activation dynamics through a unitless factor, *C*_*N*_, which quantitatively describes the rate-limiting step before the myofilaments mechanically slide across each other and generate force. Hence, in equation (1), *n *is the total number of pulses in a stimulation train,*R*_*i *_accounts for the nonlinear summation of *C*_*N *_in response to two closely spaced pulses [38], *t *(ms) is the time since the beginning of the stimulation train, *t*_*i *_(ms) is the time of the *i*th pulse in the stimulation train, and *τ*_*C *_(ms) is the time constant controlling the transient shape of *C*_*N*_.

$$\frac{dF}{dt}=A\frac{C_{N}}{K_{m}+C_{N}}-\frac{F}{\tau_{1}+\tau_{2}\frac{C_{N}}{K_{m}+C_{N}}}.$$

Equation (2) represents the development of the force recorded at the transducer due to stimulation, *F *(N), and was formulated based on a Hill-type model. This equation models the muscle as a linear spring, damper, and motor in series [24]. The development of force, *F, is *driven by *C*_*N*_/(*K*_*m *_+ *C*_*N*_), a Michelis-Menten term, which is scaled by the scaling factor of force, *A *(N/ms). In the Michelis-Menten term,*K*_*ms*_represents the sensitivity of the force development to *C*_*N*_. The second term in Equation 2 accounts for the force decay due to two time constants, *τ*_1 _and *τ*_2_. In the equation, *τ*_1 _(ms) models the force decay due to the visco-elastic components of the muscle following stimulation when *C*_*N *_is small; whereas *τ*_2_models the force decay due to these visco-elastic muscle components during stimulation.

### Research design and methods

#### Subjects

Ten individuals with hemiparesis following stroke (9 males + 1 female; age range: 46–74 years; time following stroke: 0.5–7 years) were tested (See Table 1 for subject details). All subjects signed informed consent forms approved by the Human Subjects Review Board of the University of Delaware.

**Table 1:** Detailed information about the 10 individuals with stroke tested in the study.

					Muscle Tested		
Subject #	Affected Side (Testing Side)	Age (years)	Gender	Time Post- Stroke (years)	Quadri-ceps	Dorsi-Flexor	Plantar-Flexor
1	Right	61	M	6	√	√	√
2	Right	74	M	2	√	√	√
3	Left	46	M	1.5	√	√	√
4	Left	74	M	4.5	√	√	√
5	Left	50	M	1.1	√	√	√
6	Right	57	M	1.5	√	†	√
7	Right	72	M	3.5	√	†	√
8	Right	58	F	3	*	X	√
9	Right	66	M	7	√	√	√
10	Left	65	M	0.5	*	√	*

M = Male, F = Female. (√) Indicates successfully completed data-collection. (*) Indicates that the subject's data were excluded because of inconsistent responses to stimulation for the same train within a testing session due to reflex activity, co-contraction, or the inability to relax during stimulation. (X) Indicates that measurable forces were not obtained due to excessive swelling in the subject's lower leg. (†) Indicates the subject's data were excluded due to a low signal-to-noise ratio.

#### Inclusion criteria

Subjects with no history of lower extremity orthopedic, neurological (except for stroke), or vascular problems, who had experienced a stroke at least 6-months before the testing session, were recruited for the study. All subjects were ambulatory (with or without assistive devices), had sufficient speech and cognitive abilities to understand the testing procedures and provide informed consent, and had no ankle or knee joint contractures that prevented the subjects from attaining the range of motion required for testing. The passive range of motion in the paretic limb of the subjects was adequate to enable positioning in supine with the hip and knee fully extended (0°) and the ankle positioned in neutral (0°). In addition, 14-Hz trains were delivered to to ensure that the subjects were comfortable with the sensation of stimulation and their muscles could generate recordable forces in response to electrical stimulation. No exclusions were made on the basis of gender, race, or ethnic origin.

#### Measurement procedures

Subjects were positioned on a force dynamometer (KinCom III 500-11, Chattecx Corp., Chattanooga, TN). The subjects could see a representation of the force recorded by the force transducer on a display screen. Electrical pulses were delivered using a Grass S8800 stimulator (Grass Instrument Company, Quincy, MA) with a SIU8T stimulus isolation unit. A personal computer equipped with a PCI-6024E data acquisition board and a PCI-6602 counter-timer board (National Instruments, Austin, TX) were used. A custom-written LabVIEW program (National Instruments, Austin, TX) was used for data-acquisition. The positioning on the force transducer and electrode placement varied depending on the muscle group being tested, as follows:

#### Quadriceps femoris

The testing of quadriceps muscles has been described in detail previously [28,39]. The subjects were seated on the force dynamometer with their hips flexed to approximately 75° and their knees flexed to an angle of 90°. The force transducer pad was positioned against the anterior aspect of the leg, about 5 cm proximal to the lateral malleolus. The distal portion of the subjects' thigh, waist, and upper trunk were stabilized using inelastic straps. Two self-adhesive surface electrodes (Versa-Stim 3" × 5", CONMED Corp., New York, USA) were placed on the anterior aspect of the subjects' thigh. The anode was positioned over the proximal portion of the rectus femoris and vastus lateralis; while the cathode was positioned over the distal portion of the thigh, over the vastus medialis and distal portion of the rectus femoris.

#### Ankle dorsiflexor and plantar-flexor muscles

**Figure 2:**
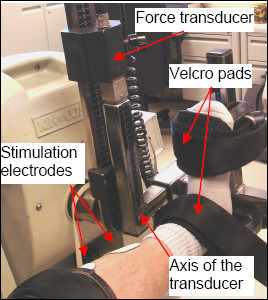
Experimental setup for testing the ankle dorsi- and plantar-flexor muscle groups.

Subjects were positioned supine on the force dynamometer with their hips extended to approximately 0° and knee fully extended (0°). The dorsiflexor muscles were tested with the ankle positioned in 15° plantarflexion and the plantar-flexors were tested with the ankle positioned at neutral position (0°). The axis of the ankle joint was aligned with the axis of the force transducer (Figure 2). The distal portion of the foot, the distal and proximal portions of the leg, and the distal portion of the subject's thigh were stabilized using inelastic velcro pads. Electrical stimulation was delivered via self-adhesive electrodes (TENS Products, Grand Lake, CO, USA; 2" × 2" Square Foam for dorsiflexor muscles; 3" Round Tricot for plantar-flexor muscles). For the dorsiflexor muscles, the cathode electrode was placed over the motor point of the tibialis anterior [40]. The anode was placed over the dorsiflexor muscle belly on the distal portion of the antero-lateral aspect of the leg; and the placement was adjusted to ensure that negligible eversion/inversion ankle moments were produced. For the plantar-flexors, the cathode was placed over the widest portion of muscle belly, covering both the medial and lateral heads of the gastrocnemius; the anode was placed over the distal portion of the gastrocnemius muscle belly.

#### Measurement protocol

Each subject participated in 1 or 2 testing sessions with at least 48 hours separating the sessions. The subjects were requested to refrain from any strenuous exercise 48 hours prior to testing. First, we familiarized the subjects with the testing procedures and ensured that they satisfied all the criteria for inclusion in the study. Following this, data were collected from the subjects' muscles. We attempted to test all 3 muscle groups during one session, with the order of muscle testing randomized across subjects. However, if the subjects were unable to complete all 3 muscle tests during the first session, a second session was performed to test the remaining muscle(s).

Stimulation trains (frequency: 14 Hz, train duration: 770 ms) of gradually increasing intensity were delivered to familiarize the subjects with the sensation of the stimulation and to confirm appropriate electrode placement. The pulse duration was maintained at a constant value of 600 μs for the entire study. Next, the stimulus amplitude was set using 500-ms long 100-Hz trains. For the quadriceps femoris muscle testing, before the stimulation amplitude was set, a series of single pulses (twitches) of gradually increasing amplitude were delivered with a rest interval of 5 seconds to obtain the subjects' maximal twitch force. For the quadriceps femoris and plantar-flexor muscle groups, the amplitude was set to either the subject's maximal tolerance or to elicit a peak force equal to twice the subject's maximal twitch force, whichever occurred first. For ankle dorsiflexor muscles, the amplitude was set to either produce a force of 60-N or to the subject's maximal tolerance, whichever occurred first. Once the stimulation amplitude was set, it was kept constant during the remainder of the session. The 100-Hz train was used to set the amplitude because this was the highest frequency used during the session. None of the trains delivered subsequently during the session would, therefore, produce greater discomfort than the 100-Hz train. The maximal twitch force was not used as a criterion to set amplitude for testing ankle dorsiflexor muscles because of problems associated with high signal-to-noise ratio due to low forces generated by single twitches.

After the stimulation amplitude was set, a series of testing trains was delivered to the muscle. First, eleven 770-ms long, 14-Hz trains were delivered to potentiate the muscle [41]. Next, a series of 40 stimulation trains of different frequencies ranging from 10 to 80-Hz and with 3 different pulse patterns (CFTs, VFTs, and DFTs) were delivered in random order at the rate of 1 train every 10 seconds, followed by the same series of 40 stimulation trains in reverse order. All the testing trains were either 1 second in duration or contained 50 pulses, whichever yielded the shorter train duration. Next, a 15 minute rest was provided before the same procedures and protocol were repeated to test the second and third muscles.

#### Identification of model parameter values

Similar procedures were used to identify the model parameter values and predicted forces for all 3 muscle groups. Preliminary tests showed that the 50-Hz CFT and 20-Hz DFT were the best pair of trains for identifying the model parameter values for all 3 muscle groups. Thus, for this study, we were able to use measured forces in response to only 2 trains to obtain all the parameter values for each subject. Because the simplest model is desirable for FES [22], we attempted to limit the number of free parameters for our force model. Preliminary analyses showed that by fixing *R*_0 _at value of 5 and *τ*_*c *_at value of 11 ms, the model accurately predicted the force responses to a range of stimulation frequencies and patterns for all the three muscle groups. Thus, the values of only 4 free parameters, *A*, *K*_*m*_, *τ*_1_, and *τ*_2_, needed to be identified for each muscle group (See Table 2 for parameter values). Parameter *τ*_1 _was calculated using the force decay following termination of the stimulation trains when *C*_*N *_approached zero (Ding et al, 2002). The remaining three parameter values (*A*, *K*_*m*_, *τ*_2_) were identified using feasible sequential quadratic programming (CFSQP) [42] to minimize the objective function *G*:

**Table 2:** Parameter Values*

**Muscle**	Subject	***A*(N/ms)**	***τ***_1_**(ms)**	***τ***_2 _**(ms)**	***K***_*m*_
**Quadriceps Femoris (N = 8)**	#1	0.351	292.7	503.1	0.067
	#2	0.412	31.2	200	0.01
	#3	2.878	72.6	1	0.215
	#4	1.153	46.9	69.2	0.016
	#5	0.53	46.1	84.7	0.012
	#6	0.682	169.3	31.1	0.034
	#7	1.504	38.591	39.58	0.016
	#9	1.04	61.8	5.5	0.024
	***Average***	***1.07***	***94.9***	***116.8***	***0.049***
	COV**	***78%***	***96%***	***144%***	***141%***

Dorsiflexor **(N = 7)**	#1	0.183	99.9	86.4	0.054
	#2	0.143	132.6	0.001	0.01
	#3	0.282	73.5	61.6	0.022
	#4	0.091	183.0	1	0.005
	#5	0.193	153.4	1	0.004
	#9	0.305	84.8	53.8	0.011
	#10	0.356	70.0	0.01	0.01
	***Average***	***0.222***	***113.9***	***29.1***	***0.017***
	COV **	***43%***	***38%***	***127%***	***101%***

Plantar-flexor **(N = 9)**	#1	0.194	373.7	1	0.231
	#2	0.28	75.0	240.6	0.033
	#3	0.963	51.7	104.6	0.017
	#4	0.287	63.6	260.1	0.013
	#5	0.187	35.5	311.6	0.02
	#6	0.287	654.3	1	0.01
	#7	0.291	67.1	259.1	0.02
	#8	0.343	115.2	91.7	0.052
	#9	0.381	55.1	132.4	0.02
	***Average***	***0.357***	***168.7***	***155.8***	***0.046***
	COV**	***71%***	***123%***	***78%***	***151%***

*Please note that for each muscle group, parameter *R *was fixed at 5 and *τ*_*c *_was fixed at 11 ms. In addition, certain muscle groups were not tested due to reflex responses or muscle swelling (see text and Table 1 for details).

** COV, Coefficient of variation = (Standard Deviation/Average) × 100

$$G(A,K_{m},\tau_{2})=\sum_{p}{\left(F^{pred}(t_{p;}A,K_{m},\tau_{2})-F^{meas}(t_{p})\right)}^{2}$$

In the above equation, *F*^*pred *^is the force predicted by equations (1) and (2) as a function of time, and depends on parameters *A*, *K*_*m*_, and *τ*_2_; *F*^*meas*^represents the force measured at time *t*_*p*_; *p *is the number of force data points. Equation (1) was solved using its analytical solution, equation (1A), and equation (2) was solved using the fourth order Runge-Kutta method. For all subjects, the optimizer was able to minimize the above objective function (Equation 3) within several seconds. Finally, the parameter values obtained using the measured forces from the 2 trains described above were used in equations 1 and 2 to obtain predicted forces for all frequencies (10 to 80-Hz) and patterns (CFTs, VFTs, and DFTs) tested. Measured versus predicted force-time responses, peak forces, and force-time integrals were compared for all trains tested except the 2 trains used to determine the model parameter values.

### Data management and analyses

**Figure 3:**
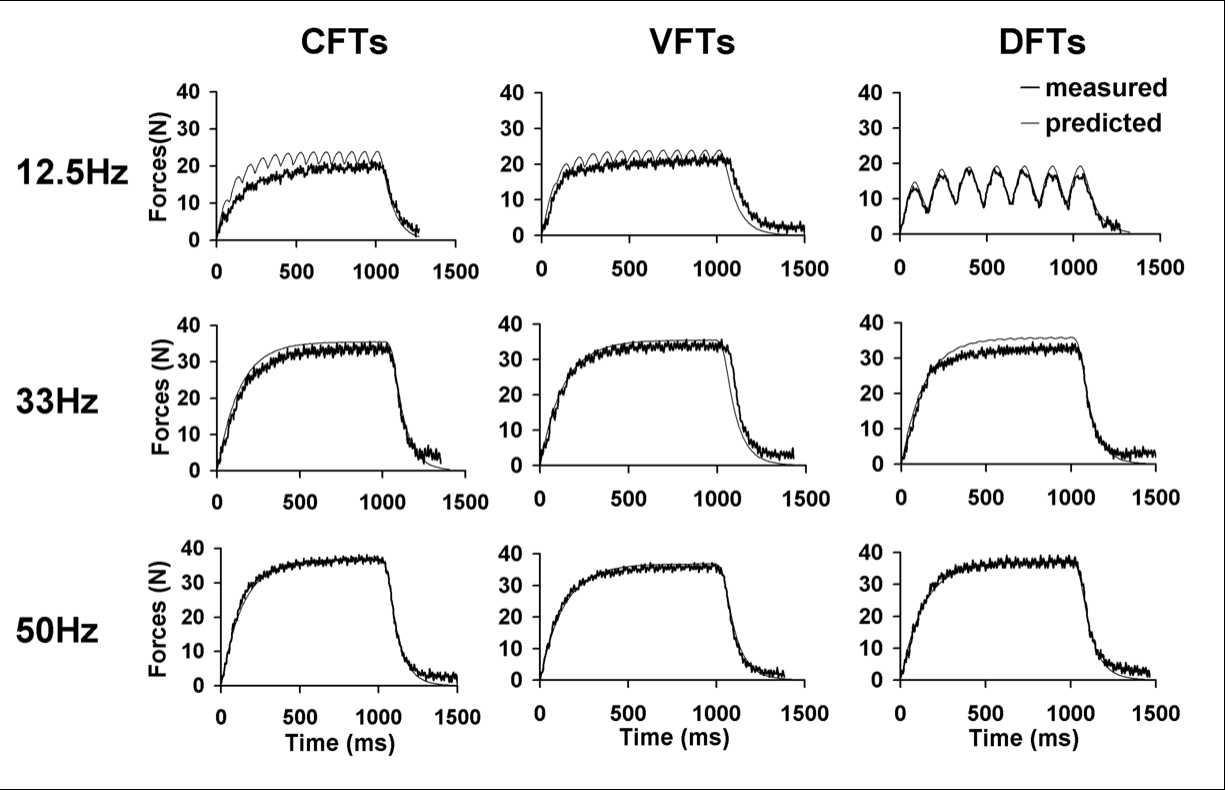
Examples of predicted and measured force responses of dorsiflexor muscles for 3 stimulation frequencies (top to bottom: 12.5, 33, and 50 Hz) and 3 different stimulation patterns (left to right: CFTS, VFTs, and DFTs).

**Figure 4:**
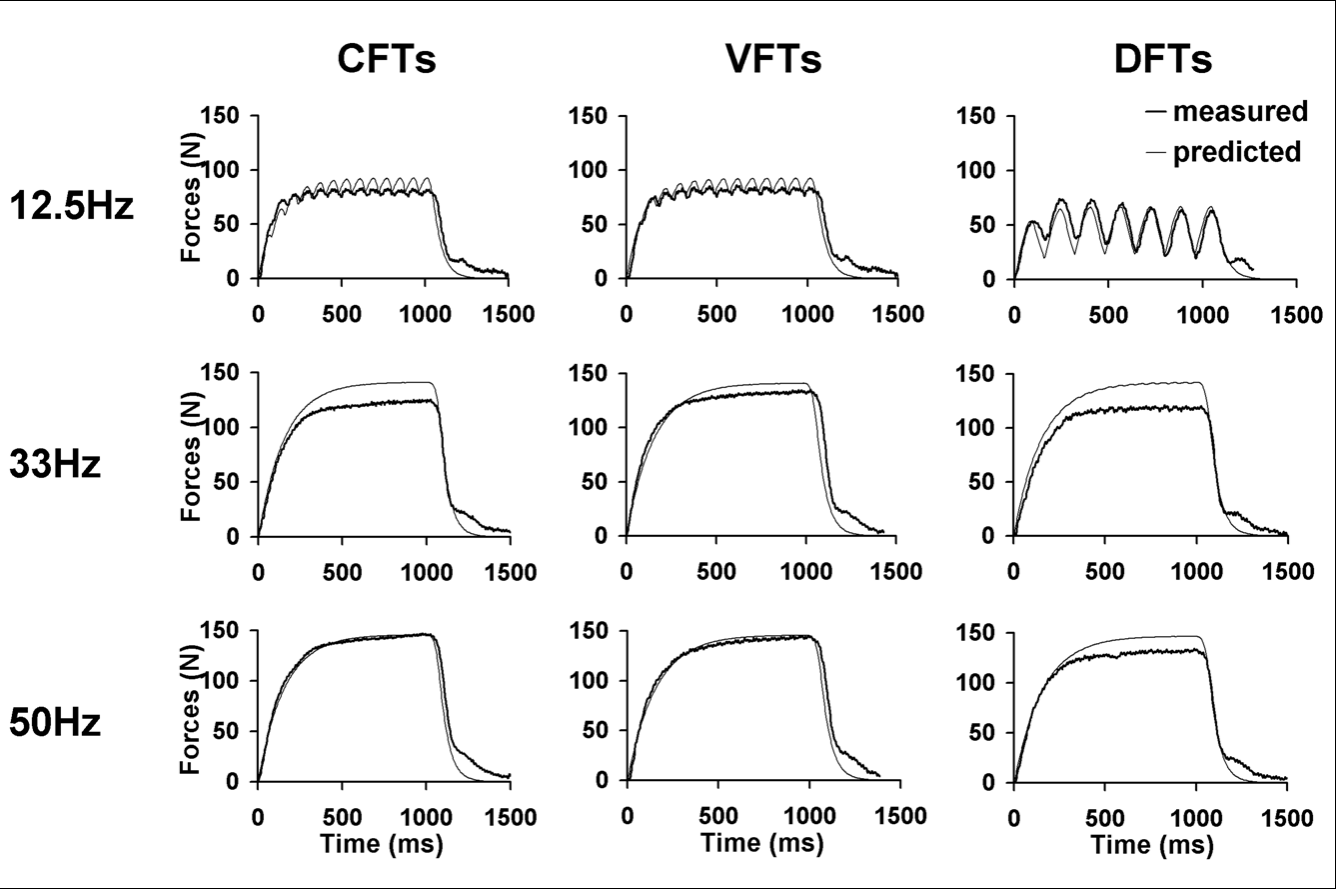
Examples of predicted and measured force responses of plantar-flexor muscles for 3 stimulation frequencies (top to bottom: 12.5, 33, and 50 Hz) and 3 different stimulation patterns (left to right: CFTS, VFTs, and DFTs). In the measured force data, note that force does not return to baseline at the end of relaxation due to the presence of reflex responses. Data shown are from the same subject whose data are shown in Figure 3.

Methods for data analyses were similar for each of the 3 muscle groups tested. For each stimulation train, the force-time responses were plotted for both the measured and the predicted forces (See Figures 3 and 4 for examples). For each subject, the force-time responses to each stimulation train were screened; we excluded data for a subject's muscle from analyses if these responses had excessive noise due to low signal to noise ratios, a lack of a one-to-one correspondence between the measured forces and each of the stimulation pulses, or the lack of clear initiation and relaxation of forces at the beginning and end of each stimulation train, respectively. For all testing trains, if both occurrences were free from excessive contamination due to presence of reflex responses, the averaged force-time responses over the two occurrences were used as the measured forces. However, if only one occurrence of a particular testing train was free from excessive contamination due to reflex responses, then that occurrence was used as the measured force. For each testing train, the force-time integrals (FTI, area under the force-time curve) and peak forces (PK, maximum instantaneous force) were calculated for both predicted and measured force-time responses.

#### Testing the model's predictive ability

Three different methods were used to test the accuracy of the model's predictions.

##### (i) Comparison of shapes of measured and predicted force-time responses

For each testing train, Pearson's coefficient of determination (r^2^) were calculated by performing a point by point comparison of the predicted versus measured forces at 5-ms intervals. The r^2 ^is an estimate of the percentage of variance in the measured data that can be accounted for by the predicted data [43]. A perfect match between the shapes of predicted and measured force-time responses for a train would yield an r^2 ^value of 1. For each of the 3 patterns tested, the averaged r^2 ^values for each frequency were used to assess how well the model predicted the shapes of the force-time responses.

##### (ii) Agreement between measured versus predicted FTIs and PKs -

The coefficients of determination cannot detect an offset between predicted and measured force-time responses. Thus, intra-class correlation coefficients (ICCs) were used to assess the agreement between the predicted versus measured FTI and PK for each of the 3 patterns tested across frequencies. The ICC is an index that provides an estimate of both consistency and average agreement between two or more data sets, while accounting for offsets in the data [43]. In addition, for each stimulation pattern tested, the measured FTI and PK values were plotted against the predicted FTI and PK values, respectively. Slopes of trendlines with the intercepts set at zero were used to evaluate how well the predicted and measured FTI and PK matched. An ICC of 1 and a trendline slope of 1 would suggest a perfect prediction of FTI and PK by the model.

##### (iii) Errors between measured and predicted FTI and PK

For each of the 3 patterns tested, the averaged PK-frequency and FTI-frequency relationships for both the measured and predicted forces were plotted for comparison. For each subject, the absolute differences between predicted and measured FTIs and PKs (*model error*) were calculated for each of the frequencies and patterns tested to quantitatively assess the accuracy of the model's predictions. In our previous work on able-bodied individuals, we showed that delivering the same train twice within a session gave a ± 15% error due to physiological variance, so we set model errors within ± 15% as the acceptable error range (Ding et al, 2002). However, preliminary testing showed that muscles of individuals with stroke showed greater variability and that the variability was different across the frequencies and patterns tested. Because a model cannot be expected to perform better than the physiological variability of muscles' responses, we used the physiological variability of our subjects' responses to the present testing to assess the model's accuracy. To obtain a measure of physiological variability for both FTIs and PKs, the absolute differences between the two occurrences of each testing train (*physiological error*) were calculated for each frequency and pattern. Thus, for each frequency and pattern tested, the average *model error *and *physiological error *values across all subjects were determined. For each pattern, if the averaged *model error *for each frequency fell within or below the 95% confidence interval of the *physiological error *for that frequency, the model's predictions were accepted as accurate.

## Results

Force responses from the quadriceps femoris, ankle dorsiflexor, and plantar-flexor muscles were measured from 10 individuals with hemiparesis following stroke (age = 62 ± 5.2 years; time post-stroke = 3.1 ± 2.1 years) (Table 1). Data from the quadriceps femoris muscles of 2 subjects and the plantar-flexor muscles of 1 subject were excluded from analyses due to the inconsistent responses during electrical stimulation because of reflex activation, co-contraction of antagonist muscles, or inability to relax during stimulation. For the dorsiflexor muscles, data from 3 subjects were excluded from the analyses due to low signal-to-noise ratios. The low force response from one of these subjects was due to swelling in the lower leg that prevented the elicitation of measurable forces (See Table 1). The model parameter values for each subject have been listed in Table 2.

**Figure 5:**
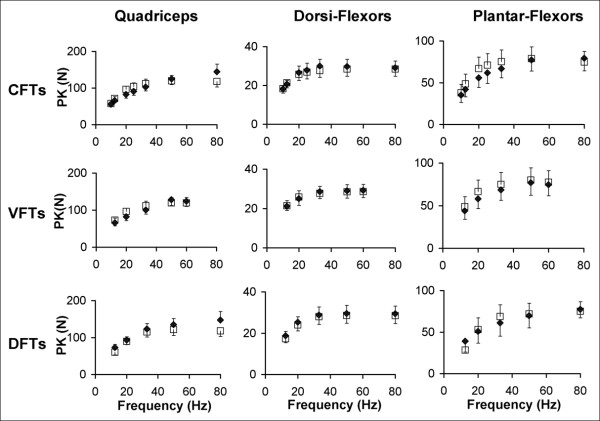
Averaged measured and predicted peak force (PK) versus frequency relationships for the quadriceps (N = 8), dorsiflexor (N = 7), and plantar-flexor (N = 9) muscles (columns: left to right) for CFTs, VFTs, and DFTS (rows: top to bottom). Error bars denote standard errors of the means.

**Figure 6:**
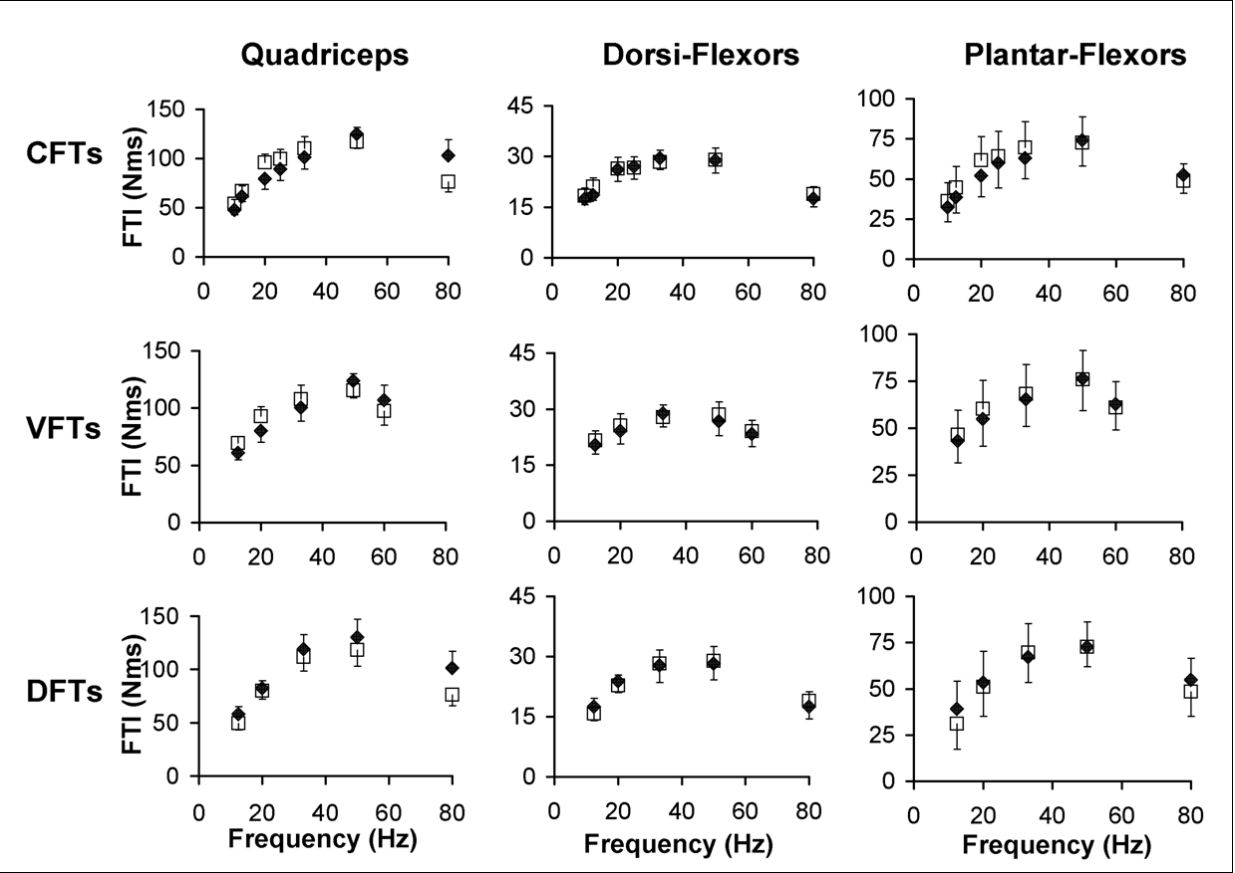
Averaged measured and predicted force-time integral (FTI) versus frequency relationships for the quadriceps (N = 8), dorsiflexor (N = 7), and plantar-flexor (N = 9) muscles (columns: left to right) for CFTs, VFTs, and DFTS (rows: top to bottom). Error bars denote standard errors of the means.

Typical measured and predicted force-time responses for the ankle dorsiflexor, and plantar-flexor muscles of a representative subject have been shown in Figures 3 and 4. Overall, the averaged FTI-frequency and PK-frequency relationships for CFTs, VFTs, and DFTs for the measured and the predicted force-time data matched well and there was consistency between the measured and predicted frequencies that generated the maximal FTI and PK for each of the muscles (See Figures 5 and 6). Interestingly, the model parameter values showed a high degree of variability across subjects and across the 3 muscles tested, with coefficients of variation ranging from 38% to 151% (Table 2).

**Figure 7:**
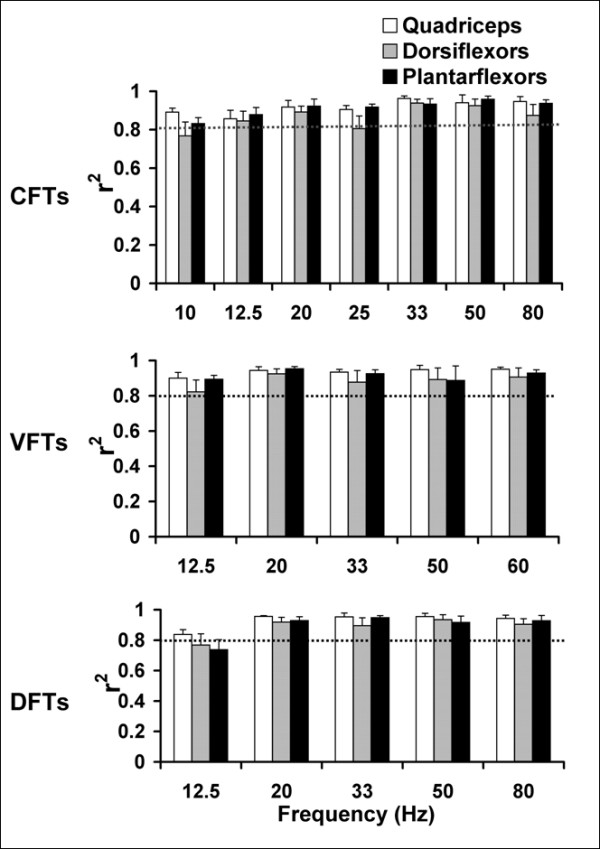
Bar graphs showing the values of the coefficients of determination (r^2^) to determine the match between the shapes of the measured and predicted force-time responses of CFTs, VFTs, and DFTs (top to bottom) for quadriceps (N = 8), dorsiflexor (N = 7), and plantar-flexor (N = 9) muscles. For each pattern and each muscle, averaged r^2 ^values and standard error bars for each frequency are plotted. The horizontal dotted line in each plot demarcates r^2 ^= 0.80.

The r^2 ^values comparing the shapes of the predicted versus measured force-time responses showed high levels of correlation between the predicted and measured forces (Figure 7). For the quadriceps muscles, the r^2^values comparing the shapes of predicted and measured force-time responses were above 0.80 for all CFTs, VFTs, and DFTs (Figure 7). For the dorsiflexor muscles, r^2 ^values were above 0.80 for all frequencies and patterns except the 10-Hz CFTs and 12.5-Hz DFTs (Figure 7). For the plantar-flexor muscles, r^2 ^values were above 0.80 for all frequencies and patterns except the 12.5-Hz DFTs (Figure 7).

**Figure 8:**
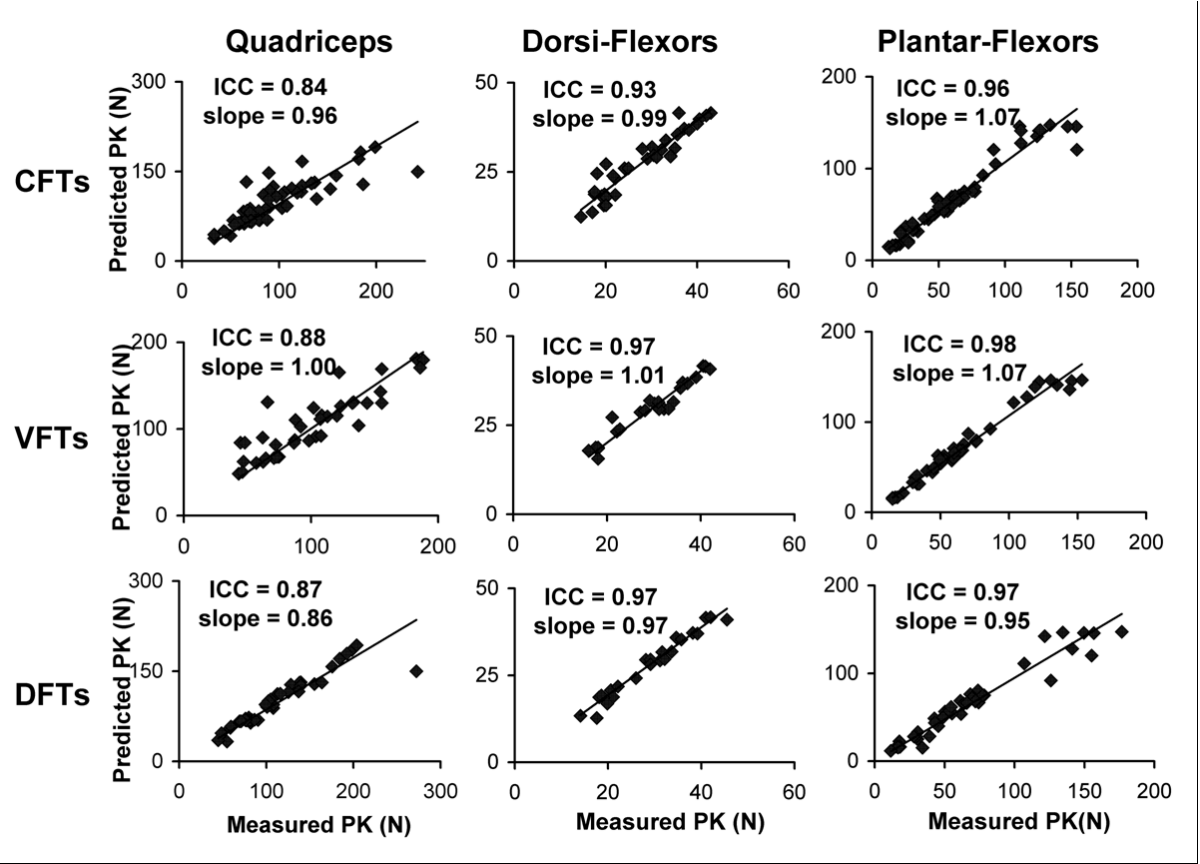
Plots of the measured versus predicted peak forces (PKs) for the quadriceps (N = 8), dorsiflexor (N = 7), and plantar-flexor (N = 9) muscles (columns: left to right) for CFTs, VFTs, and DFTs (rows: top to bottom). ICCs for agreement between measured and predicted data and the slopes of the trendlines (with intercepts set at zero) have been reported on the top left corner of each plot. A similar range of ICCs (0.82–0.97) and slopes (0.88 to 1.07) were found for agreement between the predicted and measured force-time integrals (FTIs) (not shown in figure).

ICCs comparing the measured versus predicted FTI and PK across all frequencies showed ICC values above 0.82 for the quadriceps, above 0.92 for the dorsiflexor muscles, and above 0.96 for the plantar-flexor muscles. In addition, scatter plots of predicted versus measured FTIs and PKs were plotted and the slopes of the trendlines with intercept set at zero were calculated. A perfect model would have ICC values and trendline slopes equal to one. In the current study, the trendline slopes for the 3 muscle groups tested ranged from 0.86 to 1.07 (Figure 8).

**Figure 9:**
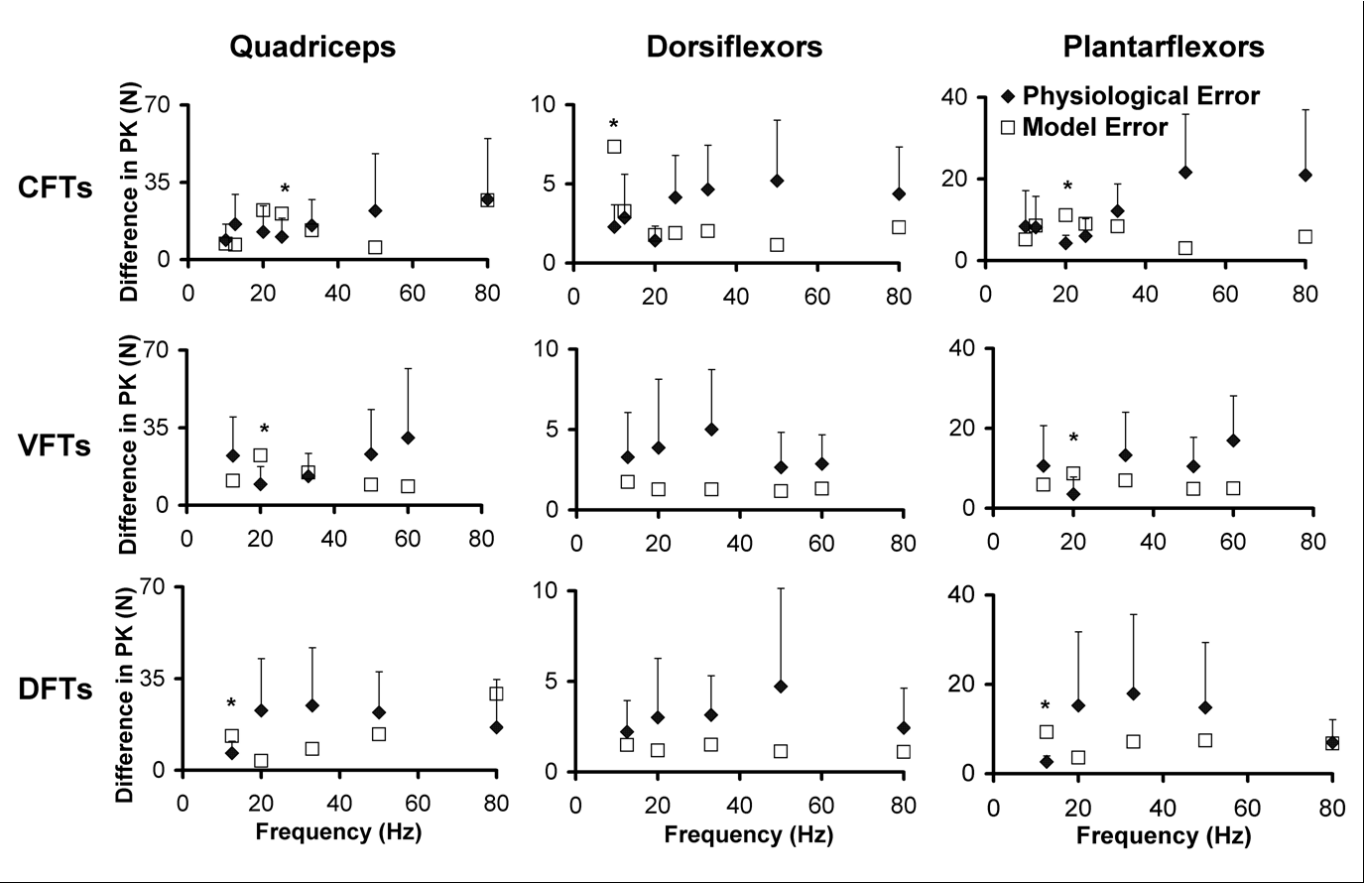
Graphs showing the model error (average absolute difference between predicted and measured PK) and physiological error (average absolute difference between 2 occurences of measured PK) along with the error bars demarcating the +95% confidence interval of physiological error for CFTs, VFTs, and DFTs (rows: top to bottom). Data for the quadriceps, dorsiflexor, and plantar-flexor muscles (columns: left to right) are shown. The model errors were considered acceptable (successful prediction) if they fell within or below the +95% confidence interval range of the physiological error. * Frequencies and patterns for which the model error was greater than the +95% confidence interval of the physiological error. Only the graphs for errors in PK are shown. Similar results were obtained for FTIs. See results for report on FTI errors.

The *model error *was within or below the +95% confidence interval of the *physiological error *for 91% of the comparisons between measured and predicted forces for the quadriceps, 94% of the comparisons for the dorsiflexor muscles, and 88% of the comparisons for plantar-flexor muscles (See Figure 9). The patterns for which the *model errors *was above the +95% confidence interval of the *physiological error *were: 25-Hz CFT PK, 20-Hz VFT PK, and 12.5-Hz DFT PK for the quadriceps; 10-Hz CFT PK and 10-Hz CFT FTI for the dorsiflexor muscles; 20-Hz CFT PK, 20-Hz VFT PK, and 12.5-Hz DFT PK and 12.5-Hz DFT FTI for plantar-flexor muscles (See Figure 9 for PK data).

## Discussion

The model accurately predicted muscle forces in response to electrical stimulation for the quadriceps femoris, ankle dorsiflexor, and plantar-flexor muscles of individuals with hemiparesis following stroke. The model successfully predicted the shape of the force-time responses (Figures 3, 4, and 5), the FTIs, and the PKs for all stimulation trains tested (Figures 5 and 6). The *model error *fell within or below the 95% confidence interval of the *physiological error *for 91%, 94%, and 88% of the comparisons between measured and predicted FTIs and PKs for the quadriceps, dorsiflexor, and plantar-flexor muscles, respectively. With only 4 free parameters, the model parameter values were first determined for each muscle using force responses to two 1-sec long stimulation trains (50Hz-CFT and 20Hz-DFT); the model was then able to predict force responses to a variety of trains of three different patterns (CFTs, VFTs, and DFTs) and a wide range of frequencies (10 to 80 Hz).

Our laboratory previously developed a mathematical model system that successfully predicted responses to electrical stimulation during both isometric [26,39] and nonisometric contractions [44,45]. The present work was the first to test the mathematical force model on the muscles of individuals with hemiparesis following stroke. Unlike the 50-Hz CFT and 12.5-Hz VFT that were used to determine the parameter values in previous studies [26,39], in the present study we fit the measured forces in response to a 50-Hz CFT and 20-Hz DFT to determine the parameter values for each subject. Previously, we have found that fixing parameter *τ*_*C *_at 20 ms for able-bodied individuals [39] and at 0.22 times each subject's half-relaxation time for individuals with spinal cord injury [26] enabled accurate predictions of isometric forces. Also, previously, parameter *R*_0 _was determined using the relationship *R*_0 _= *K*_*m *_+ 1.04 in healthy subjects [39], or set free in subjects with spinal cord injuries [26]. In this study, however, extensive preliminary testing showed that fixing *τ*_*C *_at 11 ms and *R*_0 _at 5 produced satisfactory predictions for all 3 muscles. As a result of fixing both *τ*_*C *_and *R*_0 _in the present study, the time course of *C*_*N *_was identical across muscles and subjects, and only varied with the stimulation frequency and pattern (See equation (1)). Thus, parameter *K*_*m *_was the only factor that determined the effect of *C*_*N *_on force generation for different muscles and different subjects, and equation (2) played the primary role in prediction of muscle forces. The current version of our model, therefore, has the advantage of having only 4 free parameters, only equation (2) primarily governing force predictions, and the ability to predict muscle forces for multiple muscles of individuals with hemiparesis following stroke.

Muscles of individuals with stroke show changes in histochemical, morphometric, and structural properties compared to able-bodied individuals, with most studies reporting an increased percentage of type I fibers [46-48]. In addition, in the present study, reflex responses were often observed, especially for forces measured from the quadriceps and plantar-flexor muscle groups (See Figures 3 and 5 for examples). In light of marked differences in muscle physiology following stroke [46-48] and the presence of reflex activation, a modified interpretation of the model parameter values was needed in the present study. In our previous works, all measured muscle force responses were directly in response to electrical stimulation. However, in the present study, the measured forces were often a result of the combination of synchronous activation of motor units by the electrical stimulation and the asynchronous reflex activation. Reflex activation was manifested by the lack of return of force to baseline after the stimulation was turned off, and by the lack of one-to-one correspondence between the individual pulses in the stimulation trains and the shape of the force-time responses. Furthermore, because we identified the model parameter values by fitting the measured forces, the model parameter values reflected both the effects of forces in response to electrical stimulation and any reflex responses produced. In the present study, therefore, we slightly modified the physiological interpretation of 4 of our model parameters - *C*_*N*_*,, K*_*m*_, *τ*_1, _and *τ*_2_. Previously, *C*_*N *_was defined as a unitless representation of the Ca^2+^-troponin complex; *K*_*m *_was defined as the sensitivity of strongly bound cross-bridges to *C*_*N *_; *τ*_1 _and *τ*_2_were defined as time constants of force decline in the absence and presence of strongly bound cross-bridges, respectively [39]. In the present study, we reinterpreted *C*_*N *_as the rate-limiting step before the myofilaments mechanically slide across each other and generate force; *K*_*m *_as the sensitivity of force development to *C*_*N *_; *τ*_1 _and *τ*_2 _as time constants modeling the force decay, both electrically induced and reflexive, due to the visco-elastic components of muscle in the absence and presence of stimulation, respectively. Thus, the parameter definitions in the present study were more generic and applicable to force responses of whole muscles to electrical stimulation, both in the presence and absence of reflex activation.

Unlike our previous modeling studies [26,39], in the present study we found a high degree of variability in the model parameter values within each muscle tested across the individuals with stroke, with coefficients of variation ranging between 38% and 151% (See Table 2). This variability in parameter values may be in part due to differences across subjects in the level of reflex activation of their muscles. Our model works under the assumption of synchronous activation of the whole muscle in direct response to electrical stimulation. However, in muscles of individuals with stroke, this assumption was violated due to the presence of reflex activation. Nevertheless, in spite of marked differences in properties of muscles of hemiparetic individuals and the presence of reflex activation, our model was able to predict isometric forces for 80% of the muscles studied.

A recent study comparing 3 different muscle models found that the 2^nd ^order nonlinear model developed by Bobet and Stein (1998) and our model [38], both consisting of 6 parameters, showed better predictions of muscle forces than a simple linear model, especially for higher frequency or variable frequency trains [27]. Furthermore, our model produced the least percentage error between measured and predicted among the three types of models compared [27]. In addition to the accuracy of our model demonstrated by two recent comparative studies of muscle models [27,30], the current version of the model has the added advantage of only 4 free parameters. Interestingly, in a recent sensitivity analyses of 3 muscle models, Frey Law and Shields [49] suggested that due to the influences of parameter *τ*_*C *_on muscle force properties predicted by our model, we should perhaps keep the value of parameter *τ*_*C *_free. However, both in our previous work [39] and in the present study, we found that our model accurately predicted muscle forces despite fixing *τ*_*C*_. Furthermore, during the preliminary testing of the model in the present study, we found that for all 3 muscles, the model predicted FTIs and PKs with greater accuracy when *τ*_*C *_was fixedat 11 and *R*_0 _was fixed at 5, compared to either when both *τ*_*C *_and *R*_0 _were free or when *τ*_*C *_was fixed at 20 and *R*_0 _was fixed at 2 (values employed for able-bodied subjects) [25,39]. Law and Shields suggested that interactions between parameters are likely to exist [49], hence we hypothesize that the parameters *τ*_1_, *τ*_2_, and *Km *were able to compensate for the fixed *τ*_*C *_. Because the fewest parameters are desirable for an ideal feedforward model in FES [22], and because preliminary testing showed that fixing both *τ*_*C *_and *R*_0 _did not compromise the predictive ability of the model, we decided to reduce the number of free parameters in our model by keeping the activation dynamics (See equation 1) constant for a given frequency and pattern across subjects and muscle groups.

A practical FES system needs both a feedforward model, which designs subject-specific and task-specific stimulation patterns, and a feedback controller, which corrects errors by informing the feedforward model when changes in stimulation patterns are needed [50]. When used in conjunction with a closed-loop controller, mathematical models can allow FES stimulators to deliver patient-specific and task-specific stimulation patterns that can adapt to the actual needs of the patient in real-time [14,26]. The use of customized stimulation patterns can reduce the energy expenditure and improve the speed at which functional tasks are performed during FES [14,22,26]. A model with the fewest parameters that can accurately predict the PK and FTI in response to a wide range of frequencies and patterns is desirable for a feedforward model in FES-systems [22,50]. The present model can accurately predict forces of 3 different muscles, which are important muscles for ambulation [31,32] and are commonly impaired in individuals with post-stroke hemiparesis [33-37]. Thus, because the present model can predict accurately and consists of only 4 free parameters, it has potential for use as a feedforward model in FES-systems for individuals with hemiparesis following stroke.

However, functional activities of daily living such as grasping, standing, and walking are composed of both isometric and dynamic contractions. The present study takes an incremental step by testing the model's predictive ability for isometric contractions in 3 different lower extremity muscles of post-stroke individuals. Before our model can be used for FES applications, we must enhance our model to incorporate the effects of stimulation intensity [51,52] and include non-isometric contractions [45]. Future work will enhance the current model into a force and motion model that can predict muscle performance during non-isometric contractions in response to a range of stimulation frequencies, intensities, and patterns, thereby facilitating the model's use in wider range of FES applications. Another limitation of the current approach is that we were unable to reliably collect the force responses to the two 1-second long trains (50Hz-CFT and 20Hz-DFT) needed to determine the subject-specific model parameter values for 20% of the muscles tested (See Table 1), either because of excessive reflex activation (10% muscles) or weak force generation (10% muscles). In a previous study on subjects with spinal cord injury, we anesthetized the skin underlying the electrodes during testing to prevent contamination of the measured force data in response to electrical stimulation with reflex responses [26]. We have not tested this approach on individuals with post-stroke hemiparesis. For the problem of low signal-to-noise ratios due to weak force generation (e.g., dorsiflexor muscles in our study), the sensitivity of the apparatus used to record forces must be improved.

## Conclusion

Our force model accurately predicted force-time responses, peak forces, and force-time integrals in response to electrical stimulation with a range of stimulation frequencies and 3 different stimulation patterns for the quadriceps femoris, ankle dorsiflexor, and plantar-flexor muscles of individuals with hemiparesis following stroke. Future work will enhance the model to predict the effects of stimulation intensity, frequency, and pattern during non-isometric movements so that the model can be incorporated into the feed-forward component of an FES controller to identify stimulation parameters required to produce an FES-task. In a practical FES system, the feedback controller can correct the stimulation output to account for errors due to time-varying phenomena such as reflex responses and muscle fatigue

## Abbreviations

FES: Functional electrical stimulation; CFTs: Constant-frequency trains; VFTs: Variable-frequency trains; DFTs: Doublet-frequency trains; FTI: Force-time integral; PK: Peak force.

## Competing interests

The authors declare that they have no competing interests.

## Authors' contributions

TMK was involved with subject recruitment, data-collection, statistical analysis, and manuscript preparation. JD was involved with data-collection, analysis, and mathematical modeling of muscle forces. RP assisted with mathematical modeling, interpretation of model parameters, and manuscript preparation. RM assisted with mathematical modeling and interpretation of model parameters. SABM and ASW supervised the design and coordination of the study and manuscript preparation. All authors read and approved the final manuscript.
